# Bioelectrical Methane Production with an Ammonium Oxidative Reaction under the No Organic Substance Condition

**DOI:** 10.1264/jsme2.ME21007

**Published:** 2021-06-17

**Authors:** Ha T.T Dinh, Hiromi Kambara, Yoshiki Harada, Shuji Matsushita, Yoshiteru Aoi, Tomonori Kindaichi, Noriatsu Ozaki, Akiyoshi Ohashi

**Affiliations:** 1 Department of Civil and Environmental Engineering, Graduate School of Advanced Science and Engineering, Hiroshima University, 1–4–1, Kagamiyama, Higashi-Hiroshima, Hiroshima 739–8527, Japan; 2 Faculty of Environment, Ho Chi Minh City University of Natural Resources and Environment, 236 Le Van Sy, 1 Ward, Tan Binh District, Ho Chi Minh City, Vietnam; 3 Agricultural Technology Research Center, Hiroshima Prefectural Technology Research Institute, 6869, Hara, Hachihonmatsu, Higashi-Hiroshima, Hiroshima 739–0151, Japan; 4 Program of Biotechnology, Graduate School of Integrated Sciences for Life, Hiroshima University, 1–3–1, Kagamiyama, Higashi-Hiroshima, Hiroshima 739–8530, Japan

**Keywords:** ammonia oxidation, bio-electricity, denitrification, methane production, microbial community

## Abstract

The present study investigated bioelectrical methane production from CO_2_ without organic substances. Even though microbial methane production has been reported at relatively high electric voltages, the amount of voltage required and the organisms contributing to the process currently remain unknown. Methane production using a biocathode was investigated in a microbial electrolysis cell coupled with an NH_4_^+^ oxidative reaction at an anode coated with platinum powder under a wide range of applied voltages and anaerobic conditions. A microbial community analysis revealed that methane production simultaneously occurred with biological denitrification at the biocathode. During denitrification, NO_3_^–^ was produced by chemical NH_4_^+^ oxidation at the anode and was provided to the biocathode chamber. H_2_ was produced at the biocathode by the hydrogen-producing bacteria *Petrimonas* through the acceptance of electrons and protons. The H_2_ produced was biologically consumed by hydrogenotrophic methanogens of *Methanobacterium* and *Methanobrevibacter* with CO_2_ uptake and by hydrogenotrophic denitrifiers of *Azonexus*. This microbial community suggests that methane is indirectly produced without the use of electrons by methanogens. Furthermore, bioelectrical methane production occurred under experimental conditions even at a very low voltage of 0.05‍ ‍V coupled with NH_4_^+^ oxidation, which was thermodynamically feasible.

Methane is the prime component of natural gas and is widely utilized as an energy source worldwide. It is mainly produced by biological and physical actions that collectively contribute to 20–80% of natural gas reserves (Rice and Claypool, 1981). Methane is physically produced through the thermal decomposition of organic matter in association with the formation of coal, gas, and oil (Schoell, 1988). Conversely, biological methane formation is primarily performed by methanogenic microbes (methanogens) in anaerobic environments (Whiticar *et al.*, 1986; Whiticar, 1999). Only methanogenic archaea are known to act as methanogens and use substrates produced from organic matter during fermentation, such as acetate, formate, and hydrogen gas. This methane fermentation occurs in nature, but has also been applied as an eco-friendly wastewater treatment technology (Onodera, 2013; Townsend-Small *et al.*, 2016). Artificially produced biogas may be utilized as an energy source after purification.

It is possible to generate electricity from organic substances. Microbial fuel cell (MFC) technology and its application to wastewater treatment have been extensively examined (Logan *et al.*, 2006; Sarmin *et al.*, 2019; Wang *et al.*, 2020). Conversely, in microbial electrosynthesis systems (MESs), methane is produced by providing electricity (Rabaey and Rozendal, 2010; Eerten‐Jansen *et al.*, 2012). High methane production is expected when MESs are applied to wastewater treatment because of the combination of methane fermentation using organic substances and the conversion of CO_2_ to methane by microbes through electricity (Clauwaert *et al.*, 2008; Clauwaert and Verstraete, 2009; Zhao *et al.*, 2016; Park *et al.*, 2018; Peng *et al.*, 2019). Ding *et al.* (2016) identified 0.8‍ ‍V as the optimal applied voltage for appropriate wastewater treatment and maximum methane production using an MES.

In the MES, bioelectrical methane production is performed without organic substrates (Cheng *et al.*, 2009; Villano *et al.*, 2010; Zhen *et al.*, 2015). Cheng *et al.* (2009) reported that carbon dioxide was reduced to methane at a biocathode potential of <–0.7‍ ‍V (vs. Ag/AgCl). At –1.0‍ ‍V (vs. Ag/AgCl), the electron capture efficiency of methane production was 96%. Two mechanisms have been proposed for biological methane production using a biocathode. At high applied voltages, methane may be produced by hydrogenotrophic methanogens using abiotic H_2_ formed in water oxidation (Wagner *et al.*, 2009; Eerten‐Jansen *et al.*, 2012). In this case, H_2_ is an important intermediate for methane production. The second mechanism is direct electrotrophic methane production. Cheng *et al.* (2009) reported that some methanogens must use electrons with CO_2_ to directly produce methane, without hydrogen as an intermediary. Previous studies on extracellular electron transfer demonstrated that applied voltage may not be effective at promoting methane production, suggesting a pathway without H_2_ (Rotaru *et al.*, 2013; Lohner *et al.*, 2014; Holmes *et al.*, 2017; Lee *et al.*, 2017). However, there are insufficient experimental data to prove direct electrotrophic methane production. It currently remains unclear whether bioelectrical methane production occurs via direct and/or indirect reaction(s) in MESs.

The CO_2_ reduction potential to methane E^0^’_cat_ at the biocathode is –0.24‍ ‍V (vs. SHE) under the standard condition at pH=7. When coupled with H_2_O oxidation (E^0^’_an_=0.81‍ ‍V vs SHE) at the anode, methane production in an MES occurs thermodynamically by applying more than 1.05‍ ‍V under the standard condition. If the oxidation of inorganic compounds with a lower potential (such as NH_4_^+^ oxidation to NO_3_^–^ and N_2_: E^0^’_an_=0.36‍ ‍V and –0.29‍ ‍V vs. SHE, respectively) occurs instead of H_2_O oxidation, methane may be produced at a lower applied voltage. However, MES studies have not provided sufficient information on the oxidation reaction at the anode, with experiments being conducted at relatively high voltages.

In the present study, we designed an MES experiment in which an organic substrate was not supplied, and NH_4_^+^ was added to the anode chamber to investigate whether methane production is possible even at very low applied voltages. Although the reaction of electrotrophic methane production with NH_4_^+^ oxidation to N_2_ thermodynamically proceeded even without a supply of electricity, this is the first study to report coupling to the NH_4_^+^ oxidative reaction. In addition, the microbial community was analyzed to identify the organisms involved in bioelectrical methane production.

## Materials and Methods

### MES set-up

The MES used in the present study consisted of two glass chambers, each with an effective volume of 70‍ ‍mL, which were connected by a 10-cm salt bridge containing 2% (w/w) agar (KF-30; Fujirika) and 20% (w/w) KCl (Fig. S1). The top of each chamber was connected to a 10-mL loss-of-resistance glass syringe to release the pressure generated in the chamber by the gas produced and also facilitate gas collection. A 9-cm^2^ electrode of carbon cloth (Toyobo) was installed in both chambers. The biocathode and anode electrodes were connected to a DC power supply (Array 3600 Series; T&C Technology) using a platinum wire. A 100-Ω resistor was inserted between the power supply and biocathode electrode to estimate the electric current by measuring voltage using a digital multimeter (FlePow; Levin Japan). Even if the external resister was inserted, the effect on the actual applied voltage was negligible when the internal resistance of the MES was high. A small amount of anaerobic sludge taken from a laboratory-scale upflow anaerobic sludge blanket (UASB) reactor was inoculated on the surface of the cathode electrode. Platinum powder (10% by weight of platinum on carbon powder; E-TEK, C-1 10% Pt on Vulcan XC-72) was coated on the surface of the anode, as described in previous studies (Müller and Spitzer, 1905; Nutt and Kapur, 1968; De Vooys *et al.*, 2001; Bunce and Bejan, 2011; Li *et al.*, 2017).

### MES operation

The MES was operated in the batch-processing mode at 30°C in a thermostatic chamber. The anodic and biocathodic chambers were filled with the same medium without organic substances and deoxidized through a nitrogen purge. The medium was composed of NaHCO_3_ (200‍ ‍mg L^–1^), NH_4_Cl (190‍ ‍mg L^–1^), NaH_2_PO_4_ (17‍ ‍mg‍ ‍L^–1^), and Na_2_HPO_4_ (124‍ ‍mg L^–1^), as well as trace elements, including FeSO_4_·7H_2_O (7‍ ‍mg L^–1^), CoCl_2_·6H_2_O (1.7‍ ‍mg L^–1^), ZnSO_4_·7H_2_O (1.5‍ ‍mg L^–1^), HBO_3_ (0.6‍ ‍mg L^–1^), MnCl_2_·4H_2_O (4.2‍ ‍mg·L^–1^), NiCl_2_·4H_2_O (0.4‍ ‍mg L^–1^), CuCl_2_·2H_2_O (0.27‍ ‍mg L^–1^), and Na_2_MoO_2_·2H_2_O (0.25‍ ‍mg L^–1^), at a pH of 7.5. The medium was completely replaced at intervals of 3, 5, 6, and 13‍ ‍d, with the batch experiment being repeated 20 times over 110‍ ‍d of operation. Each batch duration time was determined according to gas production for gas sampling. Each batch experiment was performed at a constant applied voltage in the range of 0.05–3.0‍ ‍V to investigate whether methane production is possible even at low voltages. After setting up the MES, a voltage was immediately supplied to enhance microbial activity at the biocathode, and the anode was unsterilized.

### Sampling and analyses

The volume of gas production in the respective chambers was measured using an airtight syringe. CH_4_, N_2_, CO_2_, and H_2_ concentrations were then measured using a gas chromatograph equipped with a thermal conductivity detector (GC-TCD; Shimadzu GC-8A). NH_4_^+^, NO_3_^–^, and NO_2_^–^ concentrations in the medium were measured by ion chromatography (Shimadzu HPLC-20A) at the start and end of each batch operation. Dissolved CH_4_ and N_2_ concentrations were estimated using Henry’s law.

### Microbial community

The sludge sample at the biocathode was collected on day 110 of the last MES operation and washed with phosphate buffer. DNA was extracted using the FastDNA^®^ SPIN Kit for Soil (MP Biomedicals), according to the manufacturer’s instructions. PCR amplification of the 16S rRNA gene was performed using the primer sets‍ ‍341’F (5′-CCTAHGGGRBGCAGCAG-3′) and 805R (5′-GACTACHVGGGTATCTAATCC-3′) with KAFA HiFi Hotstart ReadyMix (Kapa Biosystems). PCR conditions were as follows: the initial denaturation of DNA at 95°C for 3‍ ‍min, followed by 25 cycles at 95°C for 30‍ ‍s, at 55°C for 30‍ ‍s, and at 72°C for 30‍ ‍s, with a final extension at 72°C for 5‍ ‍min. The PCR product was purified and sequenced by the emulsion method using Illumina/Miseq (Illumina) at Hokkaido System Science. The sequences obtained were analyzed using QIIME (v1.8.0) (Caporaso *et al.*, 2010). Operational taxonomic units (OTUs) were grouped based on a threshold value of 97% identity for DNA using the UCLUST algorithm (Edgar, 2010). These OTUs were classified using the Greengenes database (McDonald *et al.*, 2012; Werner *et al.*, 2012).

Sequence data were deposited in the DDBJ database under DDBJ/EMBL/GenBank accession number DRA011341.

## Results

### Performance of batch experiments

**Fig. 1.**
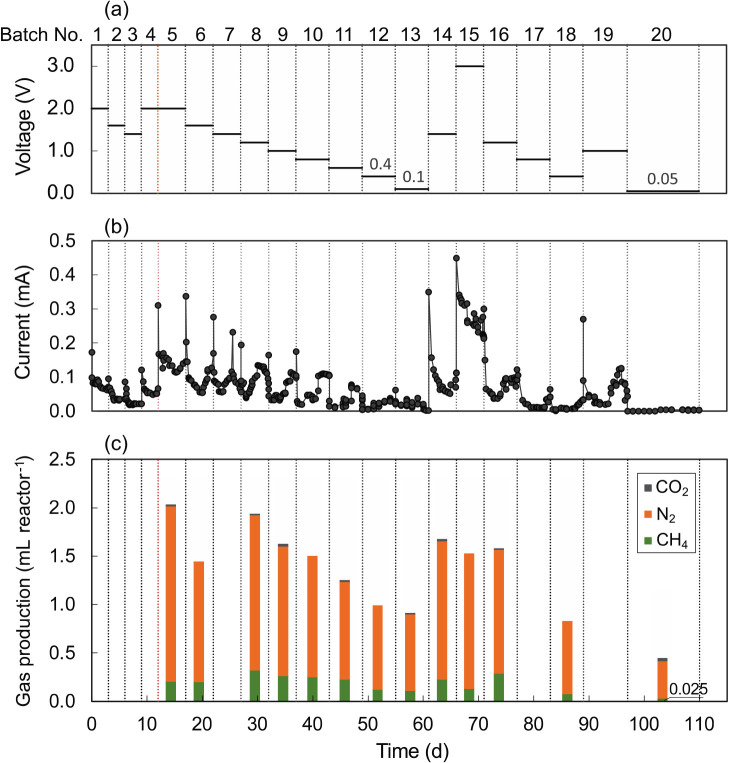
Time courses of applied voltage (a), current (b), and gases (CH_4_, N_2_, and CO_2_) produced (c) in batch experiments.

In MES batch operations, we initially attempted to apply a relatively high voltage of 2.0‍ ‍V over 3 d. Once a higher current of approximately 0.18 mA was observed, it immediately decreased to 0.09 mA and gradually declined over time, as shown in Fig. 1. However, no gas bubbles were visible in either the biocathode or anode chambers, despite the sludge inoculation being expected to enable methane production activity. The 3-d batch experiment was then repeated with changes in bulk liquid, but at lower voltages of 1.6‍ ‍V and 1.4 V, resulting in current behaviors that were the same as those at 2.0 V. Under these conditions, very few bubbles were observed in the biocathode chamber. In the next batch operation, bubbles were observed where the applied voltage was returned to 2.0 V. However, it was not possible to sample the gas produced because of its low volume.

Therefore, we changed the batch interval time from 3 to 5‍ ‍d from day 12 onwards, except for some special batches. More bubbles were produced in the biocathode chamber and collected as a gas in the fifth batch operation at 2.0 V. The gas produced was approximately 2.0‍ ‍mL on days 12–17 (Fig. 1). As expected, methane was detected, but its concentration was only 10.0%. The main component of the gas was N_2_, with a very low concentration of CO_2_. The current was markedly higher than that in the previous batch experiment at the same voltage. The current also decreased for approximately 3‍ ‍d, but increased thereafter. A significant difference was observed in current behavior between the small and large gas production chambers. In control batch experiments without the inoculation, methane was not detected in the range of 1.0–2.0 V; however, hydrogen production was observed at an applied voltage higher than 1.2‍ ‍V in the cathode chamber. Methane production is expected to be derived from inorganic carbon in the presence of microbes on the carbon cloth, the biological activity of which may be enhanced after 17‍ ‍d of operation; however, no bubbles were observed in the anode chamber under any of the conditions used.

To investigate the effects of voltage on methane production, experiments were continuously performed while decreasing the applied voltage step-by-step down to 0.1‍ ‍V until day 61 (Fig. 1). The current slightly decreased with voltage reductions; however, its pattern of behavior was similar in each batch period. Gas containing CH_4_ and N_2_ was produced at any voltage, except during days 22–27 when gas sampling failed.

Since high N_2_ concentrations of approximately 75 to 90% were detected, we reconducted batch experiments under almost identical conditions over a range of 0.05–3.0‍ ‍V on days 55–110 to reveal the source of N_2_ yield by measuring ammonium, nitrate, and nitrite. In the last batch operation, we also attempted methane production at a very low voltage of 0.05 V. A small amount of gas containing 6.09% CH_4_ was collected, even at the lowest voltage, particularly over a prolonged period of 13 d. NH_4_^+^ and NO_3_^–^ concentrations decreased in both the biocathode and anode chambers (Fig. S2). Total nitrogen ions in the two chambers decreased in all batches, suggesting that the yield of N_2_ was derived from inorganic nitrogen ions. Regarding the nitrogen balance, a strong relationship was observed between the amount of consumed NH_4_^+^ plus NO_3_^–^ and produced N_2_ (Fig. S3). Cecconet *et al.* (2019) reported the accumulation of NO_2_^–^ and N_2_O in a biocathodic denitrification process for groundwater bioremediation. However, these intermediates in denitrification were not detected in this MES experiment. The lack of accumulation of intermediates may have been caused by the slow reaction.

### Effects of voltage on gas production

**Fig. 2.**
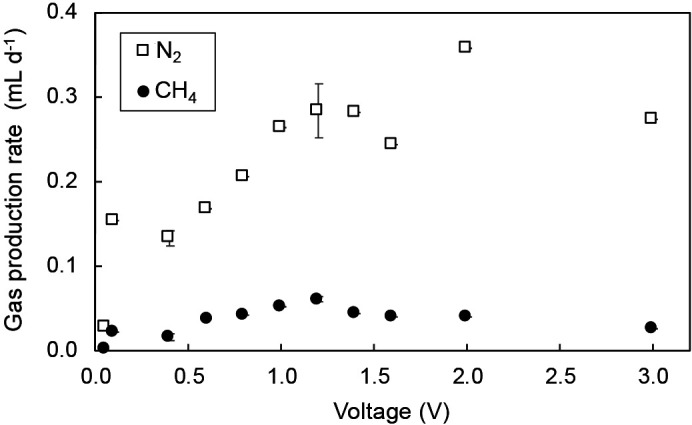
Gas production rates at different applied voltages.

Although a 100-Ω external resistor was inserted, the voltage supplied was nearly equal to the actual applied voltage between the biocathode and anode because the current versus supplied voltage was small throughout the experiment, as shown in Fig. 1. The gas production rate was significantly dependent on the applied voltage, as shown in Fig. 2. CH_4_ production slightly increased in proportion to the voltage with 0.306‍ ‍mL at 1.2 V, after which it decreased to 0.128‍ ‍mL at 3.0 V. These results suggest that a very high voltage does not always enhance methane production and may have a negative effect on microbes. The N_2_ production rate was similar to that of CH_4_ with respect to the effects of voltage; however, large fluctuations were observed. This suggests that microbes also play a role in N_2_ production. The retained microbes were expected to grow and increase with the operational time. However, they were slightly detached when bulk liquid was replaced as a result of changes in batch conditions. Therefore, the number of microbes was unstable, possibly contributing to fluctuations in gas production.

### Microbial community

**Fig. 3.**
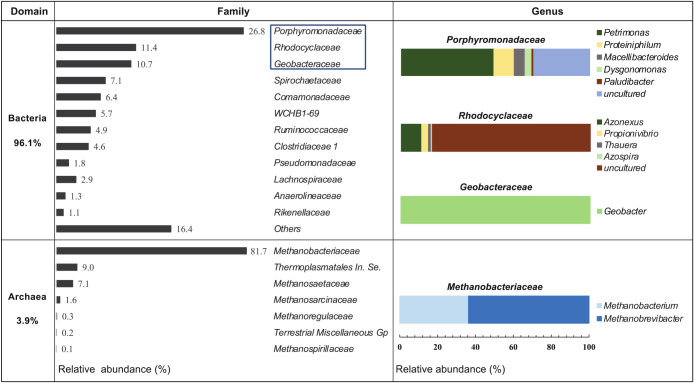
Microbial community of a biomass sample on day 110, based on the 16S rRNA gene.

In the 16S rRNA gene sequencing of the biomass sample on day 110, more than 100,000 reads, including domain bacteria and archaea, were obtained, and the number of OTUs exceeded 1,200. Sequencing results revealed the presence of bacterial and archaeal communities (Fig. 3). Archaea comprised only 3.9% of the total reads.

The major families of bacteria were *Porphyromonadaceae*, *Rhodocyclaceae*, and *Geobacterceae*, accounting for 26.8, 11.4, and 10.7%, respectively. The three families made up approximately 45% of all microbes. Of the most dominant family *Porphyromonadaceae*, approximately 50% was the obligately anaerobic genus of *Petrimonas*, while 29.8% uncultured genera were detected (Fig. 3). *Petrimonas* consists of hydrogen-producing bacteria (Lu *et al.*, 2012; Sun *et al.*, 2015; Liu *et al.*, 2016), suggesting that hydrogen is produced in the biocathode chamber. Most bacteria belonging to *Rhodocyclaceae* exhibit denitrification activity (Zhao *et al.*, 2013; Wang *et al.*, 2017). The predominant *Azonexus* genus detected, which may grow on molecular hydrogen as an electron donor (Zhao *et al.*, 2011; Liang *et al.*, 2021), plays an important role in the denitrification process to produce nitrogen gas. Only *Geobacter* was detected within the *Geobacterceae* family. The presence of electrically conductive pili or flagella on *Geobacter* species is reportedly linked to electron transfer in the MFC (Cabezas *et al.*, 2015; Yan *et al.*, 2020). In the present study, *Geobacter* appeared to be responsible for electron transfer to yield biogas.

Regarding archaea, all OTUs were *Euryarchaeota*. The majority of *Euryarchaeota* detected were methanogens, with the dominant family (81.7%) being *Methanobacteriaceae*, a hydrogen-utilizing methanogen (Fig. 3). Two genera, *Methanobrevibacter* and *Methanobacterium*, were detected at concentrations of 63.8 and 36.3%, respectively (Fig. 3). They played a major role in CH_4_ production in the biocathode chamber. In addition, *Methanosaetaceae*, an obligate acetoclastic methanogen, was detected, albeit at a low concentration (7.1%); therefore, acetate may be produced and converted to CH_4_. However, its contribution appears to have been insignificant.

The biological contributors to denitrification and methane production were identified; the produced gas containing CH_4_ and N_2_ may be explained by the presence of these microbes. Therefore, we demonstrated the biological production of CH_4_ through the provision of electricity, even at very low voltages, and in the absence of organic substances in the MES.

## Discussion

NH_4_^+^ oxidation was observed in the anode chamber, although at insignificant amounts, indicating that NH_4_^+^ was oxidized by donating electrons to the biocathode. Platinum is commonly accepted as the most promising catalyst in the electrochemical oxidation of ammonia (*e.g.*, De Vooys *et al.*, 2001; Li *et al.*, 2017). Müller and Spitzer (1905) reported that the anodic products of electrolyzing ammonia at a platinum anode were mainly NO_3_^–^ and N_2_ (25%–35%). With over-oxidation, NO_2_^–^ and NO_3_^–^ products were observed at applied voltages of higher than +0.6‍ ‍V (vs Ag/AgCl) (Endo *et al.*, 2005; Bunce and Bejan, 2011). NO_3_^–^ was also reportedly formed from NH_4_^+^ by catalytic oxidation with PtO_x_ (Fóti and Comninellis, 2004; Panizza and Cerisola, 2009). Since platinum powder was coated on the surface of the anode with a carbon cloth electrode in the present study, similar reactions to the electrolysis of water occurred; however, neither N_2_ nor NO_2_^–^ production was observed.

The oxidation of NH_4_^+^ at the anode may be represented as follows:

1/8 NH_4_^+^+3/8 H_2_O → 1/8 NO_3_^–^+5/4 H^+^+e^–^ (1)

The NO_3_^–^ produced was transferred to the biocathode chamber through the salt bridge by diffusion, resulting in a decrease in the concentration of NO_3_^–^ in the anode chamber (Fig. S3) because of N_2_ production in the biocathode chamber.

CH_4_ and N_2_ production in the biocathode chamber suggest that the reduction reactions of NO_3_^–^ and CO_2_, respectively, are represented as follows:

1/8 CO_2_+H^+^+e^–^ → 1/8 CH_4_+1/4 H_2_O (2)

1/5 NO_3_^–^+6/5 H^+^+e^–^ → 1/10 N_2_+3/5 H_2_O (3)

Based on Faraday’s laws of electrolysis, the number of donated electrons, *Ne* [mol], may be calculated from the measured current using the following equation:

$$Ne=\frac{\int Idt}{F}$$

 (4)

where *I* is current (A), *t* is time (s), and *F* is Faraday’s constant (C mol^–1^).

**Fig. 4.**
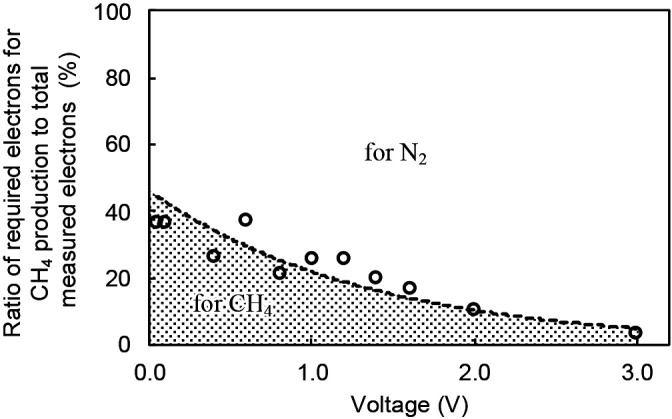
Relationship between the ratio of required electrons for CH_4_ production to total measured electrons *Ne* and applied voltage.

**Fig. 5.**
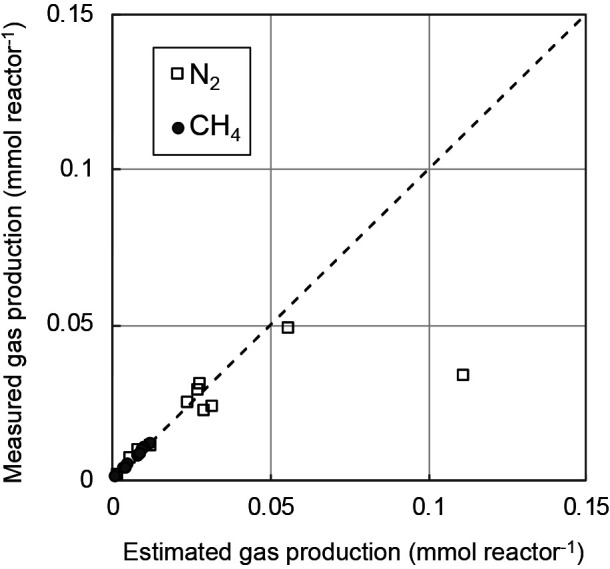
Comparison between measured and estimated gas production. An estimation of produced CH_4_ and N_2_ was performed assuming that all electrons *Ne* were used for the reduction of both CO_2_ and NO_3_^–^, while electrons from the ratio in Fig. 4 were used for CH_4_ production.

Assuming that all yield electrons *Ne*, calculated as per Eq. (4), are used for the reduction of only CO_2_ or NO_3_^–^, the amounts of CH_4_ and N_2_ produced versus the electron yield were calculated using Eqs. (2) and (3), respectively. Measured CH_4_ production was markedly less than the calculated value, while measured N_2_ production was also smaller than the theoretical value under this assumption (Fig. S4). Therefore, the two reductions were simultaneously performed, and *Ne* was distributed in both reductions. The required electrons for measured CH_4_ production from CO_2_ reduction were estimated using Eq. (2), with the ratio of required electrons to total measured electrons *Ne* shown in Fig. 4. The electron ratio slightly decreased with the applied voltage, rather than remaining constant. At very low voltages of 0.05 and 0.1 V, approximately 40% of the current was used for CO_2_ reduction to CH_4_, while only approximately 5% was utilized at 3 V. Assuming that the current to electron ratio was used for CO_2_ reduction and that the remaining electrons were used for NO_3_^–^ reduction to N_2_, as per Eq. (3), it is possible to estimate CH_4_ and N_2_ production from *Ne*. Fig. 5 compares measured and estimated CH_4_ and N_2_ production, with the curve showing the relationship between the electron ratio and voltage in Fig. 4 used in the calculation. A good agreement was observed for both CH_4_ and N_2_ production, meaning that the electron balance was almost maintained in this experiment, and the production of CH_4_ and N_2_ may theoretically be performed in the biocathode chamber according to the reduction reactions of Eqs. (2) and (3). However, at a high voltage of 3.0 V, the calculated value of produced N_2_ was markedly greater than the measured value (Fig. 5), indicating that some electrons were used for other reductions by chemical and/or microbial reactions. If NO_3_^–^ reduction to NH_4_^+^ instead of N_2_, which is the reverse reaction at the anode, is performed at the biocathode at high voltages, the reversible reactions will lead to a waste of electrons yielded in the MES. A previous study reported that a high imposing voltage exerted a negative effect on methanogens (Ding *et al.*, 2016) and nitrate-reducing bacteria (Li *et al.*, 2001; Ding *et al.*, 2016), and excessive voltage not only inhibited microbial activity, but also induced chemical reactions.

Hydrogen was not detected. However, hydrogen was expected to be produced in the biocathode chamber because of the presence of hydrogenotrophic methanogens, such as *Methanobacterium* and *Methanobrevibacter*, and the hydrogenotrophic denitrifiers of *Rhodocyclaceae (Azonexus)*. Furthermore, the hydrogen-producing bacteria *Petrimonas* were present. Previous studies on MESs also detected hydrogenotrophic methanogens, such as *Methanobrevibacter*, *Methanocorpusculum*, and *Methanoculleus sp.* (Sasaki *et al.*, 2011; Van Eerten-Jansen *et al.*, 2013; Jiang *et al.*, 2014; Siegert *et al.*, 2015). Cheng *et al.* (2019) reported that *Methanobacterium palustre* methanogens directly use electrons to produce methane without organic substances. However, this study did not provide sufficient evidence of electron utilization. Although *Geobacter* species are well-known to have the ability to transfer electrons, it was surprising that the dominant genus identified in this study was *Petrimonas* due to the lack of available information on the electron transfer ability of this genus. However, *Petrimonas* may accept electrons to produce hydrogen, which may be provided to the detected hydrogenotrophic methanogens and denitrifiers in the absence of an organic substrate in the reactor. During the bio-electrochemical production of hydrogen, it is reasonable to assume that a very small amount of hydrogen is electrochemically formed and biologically consumed. However, this electrochemical pathway may only negligibly contribute to production because hydrogen-producing *Petrimonas* was dominant in the microbial community.

Hydrogenotrophic methanogens and denitrifiers compete for the shared substrate of H_2_ produced at the biocathode. Denitrifiers are dominant in wastewater treatments under anoxic conditions in the presence of nitrate; this phenomenon may be explained by Gibbs free energy. The energy obtained in the denitrification reaction of Eq. (3) is markedly larger than that in the methane production reaction of Eq. (2). However, under hydrogenotrophic conditions, methanogens and denitrifiers were both enriched even though denitrification dominated throughout the experiment. At the lowest applied voltage of 0.05 V, approximately 40% of the H_2_ produced was utilized for methane production by the methanogens. However, with an increase in the applied voltage, the utilization ratio decreased (Fig. 4), indicating that the applied voltage affected the utilization of H_2_ by methanogens and denitrifiers. H_2_ production and concentrations are expected to increase at higher voltages. Microbes with a high affinity for substrates generally consume substrates faster than those with low affinity. The Monod constant K_m_ for H_2_ uptake was reportedly 1 and 2‍ ‍μM for *Methanobacterium ruminatium* (Lovley and Goodwin, 1988) and *Methanobrevibacter formicium* (Schauer and Ferry, 1980), respectively. In contrast, Smith *et al.* (1994) reported that the K_m_ of hydrogenotrophic denitrifiers ranged between 0.3 and 3.32‍ ‍μM. If methanogens had lower K_m_ than the denitrifiers at the biocathode, indicating a higher affinity for H_2_ and lower maximum H_2_ uptake rate, the phenomenon of a decreasing current ratio in methane production with an increasing applied voltage, as shown in Fig. 4, may be explained by this difference in K_m_ between methanogens and denitrifiers.

**Fig. 6.**
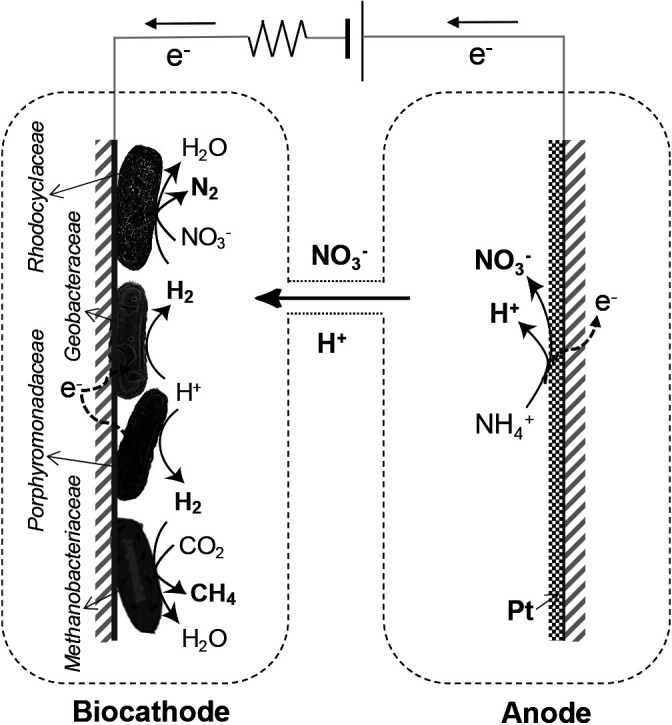
Scheme of electronic methane and nitrogen production in MES without organic substances.

Based on the experimental results obtained, Fig. 6 proposes a scheme for the process of electronic methane production used in the present study, without organic substances in the MES. Ammonium is oxidized to nitrate by a Pt catalyst at the anode with electron release. The nitrate formed is transferred into the biocathode chamber through the salt bridge. At the biocathode, the hydrogen-producing bacteria *Petrimonas* biochemically produce H_2_ by accepting electrons and protons. The H_2_ produced is biologically consumed by hydrogenotrophic methanogens of *Methanobacterium* and *Methanobrevibacter* coupled with CO_2_ uptake, and by the hydrogenotrophic denitrifiers of *Rhodocyclaceae (Azonexus)*, with transferred nitrate reduction resulting in the production of methane and N_2_, respectively. Consequently, the overall reaction at the anode and biocathode in the MES is as follows:

1/8 CO_2_+1/3 NH_4_^+^ → 1/8 CH_4_+1/6 N_2_+1/3 H^+^+1/4 H_2_O

ΔG^0^’=–3.134 kJ mol^–1^ e^–^ (5)

Thermodynamically, this reaction proceeds under the standard condition even without the provision of external energy, such as electricity, because of the negative Gibbs free energy ΔG^0^’ value. The actual condition, for example, at an applied voltage of 0.1‍ ‍V was as follows: *p*_CH4_=0.36 atm, *p*_N2_=0.65 atm, *p*_CO2_=0.03 atm, [H^+^]=15.1×10^–5^ M, and [NH_4_^+^]=5.38×10^–3^ M. In this case, the actual Gibbs free energy ΔG (=ΔG^0^’+RT ln[K]) was estimated to have a value of –5.18 kJ mol^–1^ e^–^, suggesting that the production of methane and N_2_ is expected. Therefore, the present study revealed that even in an inorganic environment, biological methane production coupled with denitrification is possible in combination with catalytic ammonium oxidation, even at very low applied voltages <0.1 V, through the three key players of hydrogenotrophic methanogens, denitrifiers, and hydrogen-producing bacteria.

## Citation

Dinh, T. T H., Kambara, H., Harada, Y., Matsushita, S., Aoi, Y., Kindaichi, T., et al. (2021) Bioelectrical Methane Production with an Ammonium Oxidative Reaction under the No Organic Substance Condition. *Microbes Environ ***36**: ME21007.

https://doi.org/10.1264/jsme2.ME21007

## Supplementary Material

Supplementary Material
