# Supplementary material for: Bioelectrical Methane Production with an Ammonium Oxidative Reaction under the No Organic Substance Condition
Source: Microbes Environ. 2021 Jun 17;36(2):ME21007. doi: 10.1264/jsme2.ME21007 (PMC8209456; doi:10.1264/jsme2.ME21007)
Supplement: Supplementary file 1 — Supplementary Material [file 36_21007_s1.pdf]

Supplementary material

Bio-electrical Methane Production with an Ammonium Oxidative Reaction under the No Organic Substance Condition

Ha T.T Dinh[1,2], Hiromi Kambara[1], Yoshiki Harada[1], Shuji Matsushita[1,3], Yoshiteru Aoi[4], Tomonori Kindaichi[1], Noriatsu Ozaki[1], Akiyoshi Ohashi[1]

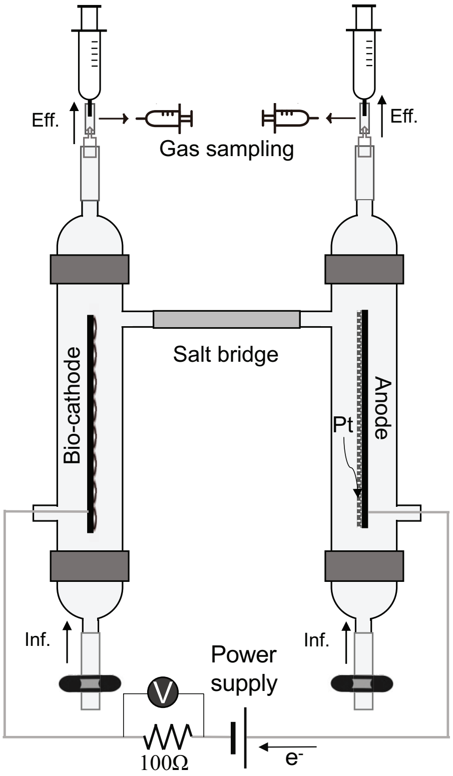

Fig. S1 Schematic of MES consisting of two chambers connected with a salt bridge. A carbon cloth electrode was installed in both chambers. Biocathode and anode electrodes were connected to a DC power supply using platinum wire. A 100 Ω resistor was inserted between the power supply and the biocathode. A platinum powder was coated on the surface of the anode.

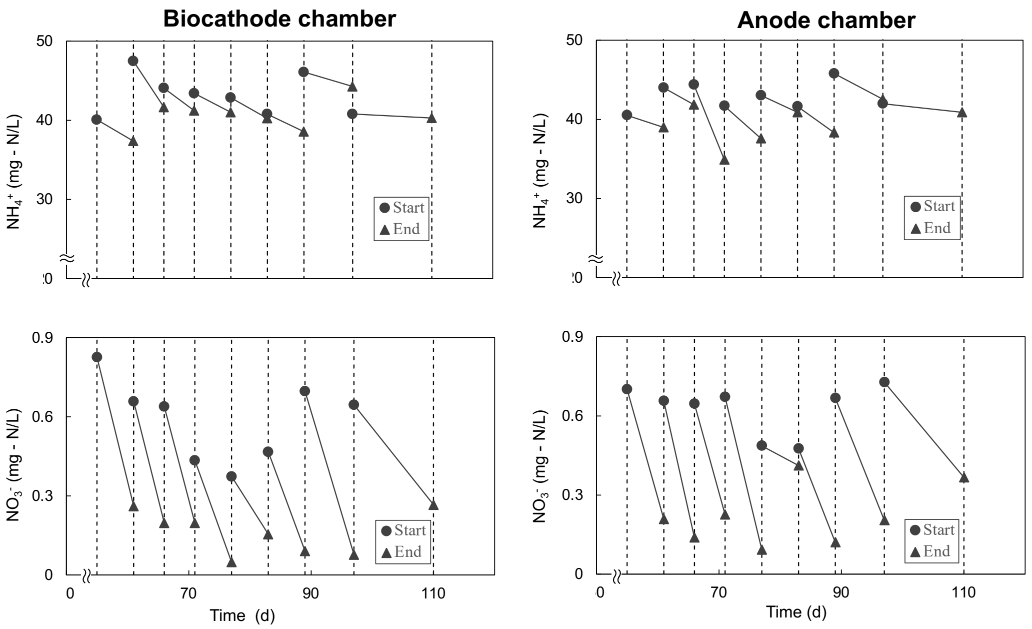

Fig. S2 NH_4^+–N and NO_3^-–N at the start and end of each batch operation.

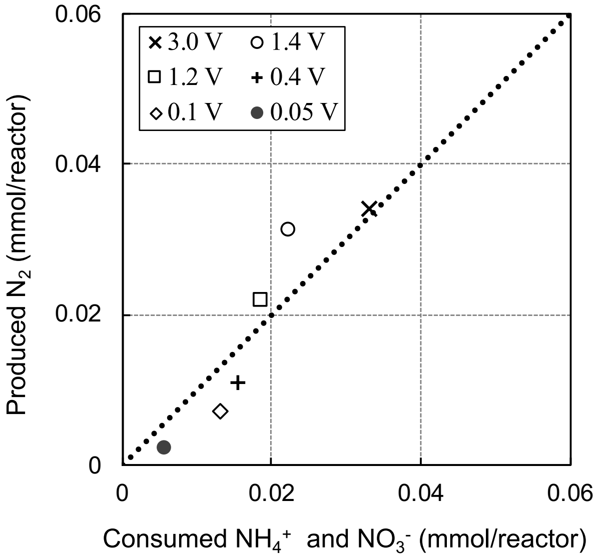

Fig. S3 Relationship between the produced N_2 and total amount of consumed NH_4^+ and NO_3^-.

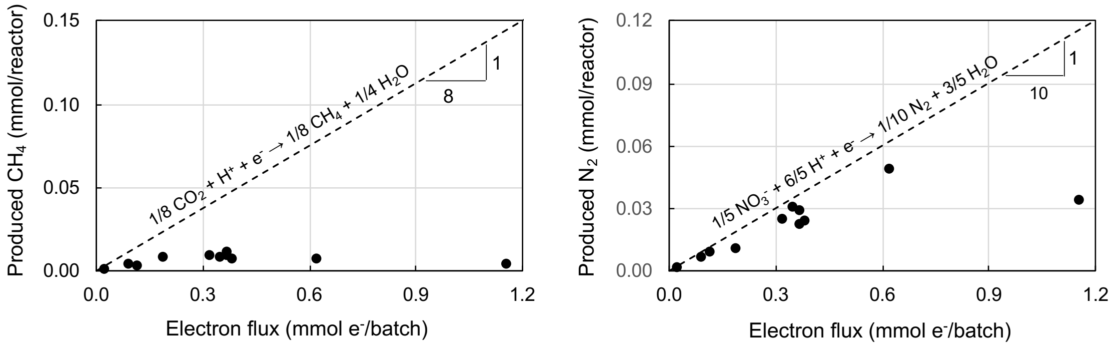

Fig. S4 Produced CH_4 and N_2 versus electron flux *Ne*. Circle: measured productions, dash lines: theoretical production assuming that all yield electrons *Ne* are only used for the reduction of CO_2 or NO_3^-.

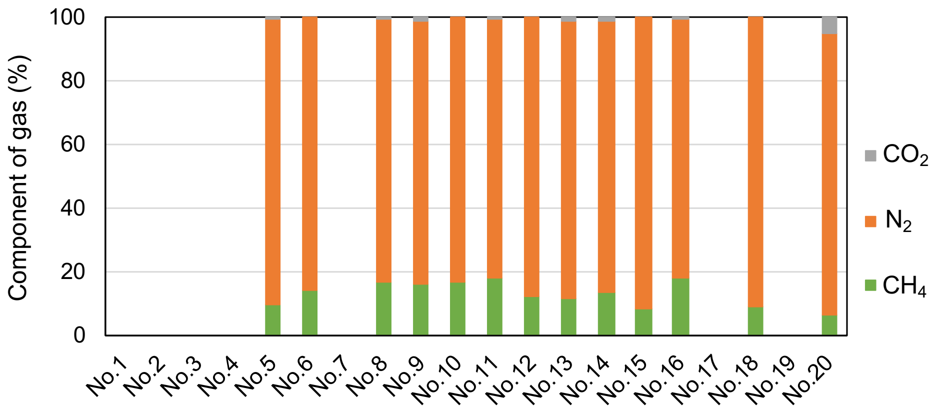

Fig. S5 Component of produced gas in the biocathode chamber.

Table S1. MES operational conditions

**Batch no.**	**Duration**	**Voltage**	**pH at the end**	
	(d)	(V)	Biocathode	Anode
1	3	2.0	8.1	6.7
2		1.6	8.1	7.5
3		1.4	7.9	7.6
4		2.0	7.9	7.4
5	5	2.0	8.6	7.0
6		1.6	8.7	7.0
7		1.4	8.5	7.0
8		1.2	7.9	7.3
9		1.0	7.6	7.2
10	6	0.8	7.6	7.1
11		0.6	7.6	7.3
12		0.4	7.4	7.3
13		0.1	7.7	7.6
14	5	1.4	7.6	7.2
15		3.0	9.7	6.8
16	6	1.2	7.7	7.2
17		0.8	7.4	7.3
18		0.4	7.5	7.4
19	8	1.0	7.6	7.2
20	13	0.05	7.5	7.5
